# An Activating Transcription Factor 5-Mediated Survival Pathway as a Target for Cancer Therapy

**DOI:** 10.18632/oncotarget.180

**Published:** 2010-10-01

**Authors:** Zhi Sheng, Sara K. Evans, Michael R. Green

**Affiliations:** Howard Hughes Medical Institute, Programs in Gene Function and Expression and Molecular Medicine, University of Massachusetts Medical School, Worcester, MA 01605, USA

**Keywords:** ATF5, anti-apoptosis, cancer therapy, malignant glioma, survival pathway

## Abstract

Genes that are highly expressed in cancer cells and are essential for their viability are attractive targets for the development of novel cancer therapeutics. Activating transcription factor 5 (ATF5) is an anti-apoptotic protein that is highly expressed in malignant glioma but not normal brain tissues, and is essential for glioma cell survival. Recent work has revealed an essential survival pathway mediated by ATF5 in malignant glioma; pharmacological inhibition of this pathway leads to tumor regression in mice. ATF5 is also highly expressed in a variety of other cancers, and preliminary studies have shown that the ATF5-mediated survival pathway is active in diverse human cancer cell lines. Targeting this pathway may therefore have therapeutic implications for the treatment of a wide range of cancers. In this perspective, we summarize recent advances in ATF5 research, focusing on its role in promoting cancer and its potential as a target for cancer therapy.

## INTRODUCTION

The mammalian activating transcription factor/cyclic AMP responsive element-binding (ATF/CREB) family of transcription factors comprises a large group of proteins whose members have diverse roles in development, differentiation, cellular proliferation and apoptosis. Members of the ATF/CREB family contain related basic leucine zipper (bZIP) domains; the basic region is enriched with lysine and arginine residues and is involved in DNA binding, whereas the leucine zipper motif mediates protein-protein interactions. ATF/CREB proteins bind as homo- or hetero-dimers to a DNA-binding site known as the cyclic AMP responsive element (CRE), which has the consensus sequence TGACGTCA (reviewed in [1]).

ATF5, previously designated as ATFx (or ATF7 in mice), was first isolated as a binding partner of granulocyte colony-stimulating factor (G-CSF) gene promoter element 1-binding protein (GPE1-BP), a protein involved in regulating G-CSF expression [2]. Subsequent studies have revealed that ATF5 can either homodimerize or form heterodimers with ATF4 or CCAAT enhancer-binding protein (C/EBP), and bind CRE elements [3,4] or a recently-identified novel DNA motif with the consensus sequence C(C/T)TCT(T/C)CCTTA [5]. ATF5 has also been shown to heterodimerize with the human T-cell leukemia virus type 1 (HTLV-1) Tax transactivator protein and bind to the Tax-responsive element, a member of the asymmetric CRE family of enhancer sequences [6].

A role for ATF5 in promoting cell survival was first suggested from gene expression profiling analysis in murine pro-B lymphocytic cells induced to undergo apoptosis following withdrawal of the cytokine interleukin-3 (IL-3) [7]. Among the most dramatic changes observed was a large decrease in *ATF5* expression, raising the possibility that ATF5 had a role in cell survival. Subsequent work revealed that ATF5 plays a critical role in antagonizing apoptosis induced by either the deprivation of IL-3 or the expression of a pro-apoptotic protein 24p3 in murine pro-B lymphocytes, or by growth factor withdrawal in HeLa cells [8].

## ATF5 EXPRESSION IN CANCER

In cancer cells, genes that induce apoptosis are often inactivated or down-regulated, whereas anti-apoptotic genes are frequently activated or over-expressed. Consistent with this paradigm, a number of studies have demonstrated that ATF5 is highly expressed in a variety of cancer cell types, whereas it is not detectably expressed in most normal human tissues (the exceptions being the liver, prostate and testis, where ATF5 is expressed at a high level [6,9]). For example, a comparison of ATF5 protein levels between normal and neoplastic samples using tissue microarrays revealed that in all malignant tissues examined—including those of the prostate, colon, endometrium, breast, ovary, pancreas, gastric, and lung—the percentage of ATF5-positive cells is significantly higher than that in normal tissues [10]. Similarly, a query of the Oncomine cancer profiling database revealed that, in general, the expression level of ATF5 is significantly higher in malignant tissues than their normal counterpart tissues [11]. The only exception appears to be hepatocellular carcinoma cells, which express lower levels of ATF5 than normal liver cells; this discrepancy may be due to epigenetic silencing of ATF5 in hepatocellular carcinoma cells through promoter methylation [12]. Notably, increased levels of ATF5 have been observed in primary brain tumors, and ATF5 expression is particularly high in glioblastoma, an aggressive form of malignant glioma [10,11].

A pair of studies has provided intriguing evidence that high ATF5 expression levels may correlate with poor prognosis in cancer patients. In one study, a retrospective analysis of 23 individuals with glioblastoma revealed that patients harboring tumors expressing high levels of ATF5 had substantially shorter survival times than those with tumors in which ATF5 expression was low or undetectable [11]. In another study, expression profiling in chronic lymphocytic leukemia (CLL) patients of known clinical outcome identified *ATF5* as a gene whose significant over-expression correlates with poor patient outcome [13].

## IDENTIFICATION OF AN ESSENTIAL ATF5-MEDIATED SURVIVAL PATHWAY IN MALIGNANT GLIOMA: THERAPEUTIC IMPLICATIONS

Inhibition of ATF5 activity, using a dominant negative form of ATF5, kills human and rat glioblastoma cells but does not affect normal cells surrounding the tumor, indicating ATF5 is selectively essential for the survival of glioblastoma cells [10]. The high expression of ATF5 in brain tumors, combined with the fact that it is selectively essential for glioma cell survival, make ATF5 an appealing potential therapeutic target for the treatment of malignant glioma. However, developing effective small-molecular inhibitors of transcription factors has proven to be challenging [14].

**Figure 1:**
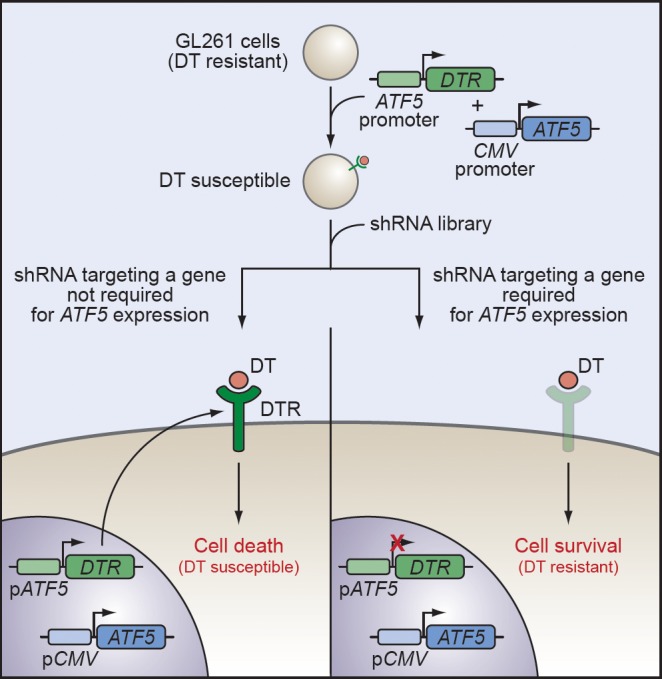
Schematic summary of the genome-wide RNAi negative-selection screen used to identify factors required for transcription of *ATF5* Diphtheria toxin (DT)-resistant mouse malignant glioma GL261 cells stably co-expressing the human diphtheria toxin receptor (*DTR*) gene driven by the mouse *ATF5* promoter and *ATF5* driven by the constitutive CMV promoter were transduced with a genome-wide mouse shRNA library. DT-resistant clones were isolated, and positive shRNAs were identified.

To uncover the upstream signaling pathways that control the expression and activity of ATF5—with the goal of identifying more targetable proteins, such as kinases, required for glioma cell survival—we performed a genome-wide RNA interference (RNAi) screen for factors that are required for transcription of the *ATF5* gene [11]. Because loss of ATF5 function within a cell would induce apoptosis, and therefore preclude the subsequent identification of candidate short hairpin RNAs (shRNAs), we developed a novel negative-selection strategy (Figure 1). This strategy was based on the ability of diphtheria toxin (DT) to kill cells that express the DT receptor (DTR). Mouse cells lack a functional DTR and are DT resistant [15]. We generated a mouse malignant glioma GL261 cell line stably expressing the human DTR driven by the mouse *ATF5* promoter; the *ATF5* promoter is normally active in GL261 cells, which drives expression of the DTR gene and confers susceptibility to DT. We then used this stable cell line to screen for shRNAs that could inactivate the *ATF5* promoter and, consequently, give rise to a DT-resistant clone. Because these shRNAs would also inhibit expression of the endogenous *ATF5* gene and induce apoptosis, the cell line was kept alive by the expression of *ATF5* driven by a constitutive promoter. DT-resistant clones were isolated, and positive shRNAs were identified and then validated for their ability to inhibit expression of the endogenous *ATF5* gene.

**Figure 2:**
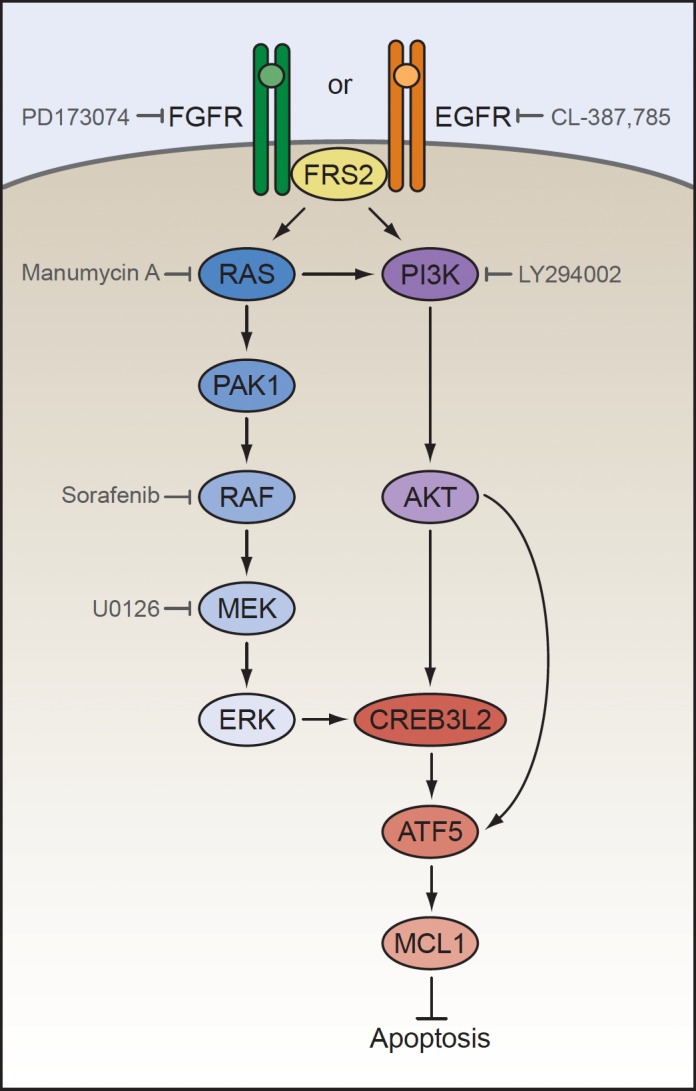
An essential ATF5-mediated survival pathway in malignant glioma Cell surface receptors (FGFR or EGFR) activate RAS/MAPK or PI3K signaling pathways through FGFR substrate 2 (FRS2) and culminate in the activation of CREB3L2, which in turn stimulates transcription of ATF5. ATF5 then antagonizes apoptosis by directly up-regulating expression of the anti-apoptotic factor MCL1. Commercially available pharmacological inhibitors of several components of the ATF5-mediated survival pathway are indicated in gray.

This approach identified 12 genes as regulators of *ATF5* expression, and further analyses revealed the upstream signaling pathways that regulate ATF5 expression in malignant glioma (summarized in Figure 2). Cell surface receptors (e.g. fibroblast growth factor receptor (FGFR) or epidermal growth factor receptor (EGFR)) activate downstream RAS/mitogen-activated protein kinase (RAS/MAPK) or phosphoinositide-3-kinase (PI3K) signaling pathways through FGFR substrate 2 (FRS2) and culminate in the activation of CREB protein 3-like 2 (CREB3L2), which in turn stimulates transcription of ATF5. ATF5 then antagonizes apoptosis by directly up-regulating expression of myeloid cell leukemia sequence 1 (MCL1), an anti-apoptotic B-cell leukemia/lymphoma 2 (BCL2) family member.

Pharmacological inhibitors of several components of that ATF5-mediated survival pathway are commercially available (see Figure 2). One of these inhibitors, sorafenib, is a multi-kinase inhibitor whose strongest activity is against RAF kinases and has been used to treat several different types of cancers [16,17]. Sorafenib inhibits the growth of human malignant glioma xenografts in mice, which suggests it may also be used to treat malignant glioma [11]. Furthermore, sorafenib sensitizes human malignant glioma xenografts to temozolomide, a chemotherapeutic agent commonly used to treat malignant glioma [18]. Inhibition of other components of the ATF5-mediated survival pathway, including FGFR (using the specific inhibitor PD173074), EGFR (CL-387,785), RAS (manumycin A), MEK (U0126), ERK (FR180204) and PI3K (LY294002), also induces apoptosis in malignant glioma cells [11]. Further investigation will verify whether these inhibitors may also be used to treat malignant glioma.

In addition to glioblastoma, ATF5 is also essential for the viability of other cancer cell types. For example, inhibition of ATF5 by a dominant negative mutant selectively kills breast cancer cells but not normal breast epithelial cells [10]. Furthermore, RNAi-mediated knockdown of ATF5 induces apoptosis in a variety of tumor cell lines derived from lung, prostate, skin, and ovary [11]. Taken together these studies demonstrate that ATF5 mediates cell survival in many cancers, and suggest that inhibition of the ATF5-mediated survival pathway may have therapeutic implications for the treatment of a wide range of malignancies.

## FUTURE DIRECTIONS

Further investigations will reveal whether the ATF5-mediated survival pathway represents an efficacious target for the treatment of cancers in addition to glioblastoma. Likewise, given that ATF5 is aberrantly over-expressed in many tumor types, it would be interesting to investigate whether high ATF5 expression is generally linked to poor prognosis of cancer patients, as has been observed in glioblastoma and CLL.

Mechanistically, a further understanding of the physiological and pathological role of ATF5 *in vivo* would greatly benefit from the generation of ATF5 knock-out mice. If homozygous deletion of the ATF5 gene does not result in embryonic lethality, then crossing ATF5 knock-out mice with mice bearing genetic mutations that induce tumorigenesis would ascertain whether ATF5 is essential for tumor formation and progression in vivo.

It is possible that ATF5 antagonizes apoptosis through multiple targets in addition to MCL1. For example, ATF5 has been shown to promote cell survival through transcriptional activation of heat shock protein 27 (Hsp27) in rat H9c2 cells [19]. Chromatin immunoprecipitation coupled with deep sequencing (ChIP-seq) could be used to identify ATF5 target genes and study ATF5 function on a genome-wide level. Identification of additional ATF5 target genes would further define the important role of ATF5 in cancer development, and may also identify additional potential therapeutic targets.

Finally, it is worth noting that the genome-wide RNAi negative-selection screening approach used to identify components of the ATF5-mediated survival pathway represents a general strategy that can be applied to identify essential survival pathways in other types of cancer cells. Such studies may reveal new regulatory pathways that contribute to malignant transformation and are potential therapeutic targets.

## References

[1] Hai T, Hartman MG (2001). The molecular biology and nomenclature of the activating transcription factor/cAMP responsive element binding family of transcription factors: activating transcription factor proteins and homeostasis. Gene.

[2] Nishizawa M, Nagata S (1992). cDNA clones encoding leucine-zipper proteins which interact with G-CSF gene promoter element 1-binding protein. FEBS Lett.

[3] Al Sarraj J, Vinson C, Thiel G (2005). Regulation of asparagine synthetase gene transcription by the basic region leucine zipper transcription factors ATF5 and CHOP. Biol Chem.

[4] Vinson C, Myakishev M, Acharya A, Mir AA, Moll JR, Bonovich M (2002). Classification of human B-ZIP proteins based on dimerization properties. Mol Cell Biol.

[5] Li G, Li W, Angelastro JM, Greene LA, Liu DX (2009). Identification of a novel DNA binding site and a transcriptional target for activating transcription factor 5 in c6 glioma and mcf-7 breast cancer cells. Mol Cancer Res.

[6] Forgacs E, Gupta SK, Kerry JA, Semmes OJ (2005). The bZIP transcription factor ATFx binds human T-cell leukemia virus type 1 (HTLV-1) Tax and represses HTLV-1 long terminal repeat-mediated transcription. J Virol.

[7] Devireddy LR, Teodoro JG, Richard FA, Green MR (2001). Induction of apoptosis by a secreted lipocalin that is transcriptionally regulated by IL-3 deprivation. Science.

[8] Persengiev SP, Devireddy LR, Green MR (2002). Inhibition of apoptosis by ATFx: a novel role for a member of the ATF/CREB family of mammalian bZIP transcription factors. Genes Dev.

[9] Pascual M, Gomez-Lechon MJ, Castell JV, Jover R (2008). ATF5 is a highly abundant liver-enriched transcription factor that cooperates with constitutive androstane receptor in the transactivation of CYP2B6 implications in hepatic stress responses. Drug Metab Dispos.

[10] Monaco SE, Angelastro JM, Szabolcs M, Greene LA (2007). The transcription factor ATF5 is widely expressed in carcinomas, and interference with its function selectively kills neoplastic, but not nontransformed, breast cell lines. Int J Cancer.

[11] Sheng Z, Li L, Zhu LJ, Smith TW, Demers A, Ross AH, Moser RP, Green MR (2010). A genome-wide RNA interference screen reveals an essential CREB3L2-ATF5-MCL1 survival pathway in malignant glioma with therapeutic implications. Nat Med.

[12] Gho JW, Ip WK, Chan KY, Law PT, Lai PB, Wong N (2008). Re-expression of transcription factor ATF5 in hepatocellular carcinoma induces G2-M arrest. Cancer Res.

[13] Mittal AK, Hegde GV, Aoun P, Bociek RG, Dave BJ, Joshi AD, Sanger WG, Weisenburger DD, Joshi SS (2007). Molecular basis of aggressive disease in chronic lymphocytic leukemia patients with 11q deletion and trisomy 12 chromosomal abnormalities. Int J Mol Med.

[14] Weiss WA, Taylor SS, Shokat KM (2007). Recognizing and exploiting differences between RNAi and small-molecule inhibitors. Nat Chem Biol.

[15] Collier RJ (1975). Diphtheria toxin: mode of action and structure. Bacteriol Rev.

[16] Favata MF, Horiuchi KY, Manos EJ, Daulerio AJ, Stradley DA, Feeser WS, Van Dyk DE, Pitts WJ, Earl RA, Hobbs F (1998). Identification of a novel inhibitor of mitogen-activated protein kinase kinase. J Biol Chem.

[17] McCubrey JA, Steelman LS, Abrams SL, Chappell WH, Russo S, Ove R, Milella M, Tafuri A, Lunghi P, Bonati A (2009). Emerging Raf inhibitors. Expert Opin Emerg Drugs.

[18] Villano JL, Seery TE, Bressler LR (2009). Temozolomide in malignant gliomas: current use and future targets. Cancer Chemother Pharmacol.

[19] Wang H, Lin G, Zhang Z (2007). ATF5 promotes cell survival through transcriptional activation of Hsp27 in H9c2 cells. Cell Biol Int.

